# Molecular Physicochemical Properties of Selected Pesticides as Predictive Factors for Oxidative Stress and Apoptosis-Dependent Cell Death in Caco-2 and HepG2 Cells

**DOI:** 10.3390/ijms23158107

**Published:** 2022-07-23

**Authors:** Amélia M. Silva, Carlos Martins-Gomes, Sandrine S. Ferreira, Eliana B. Souto, Tatiana Andreani

**Affiliations:** 1 Department of Biology and Environment, School of Life Sciences and Environment, University of Trás-os-Montes e Alto Douro (UTAD), Quinta de Prados, 5001-801 Vila Real, Portugal; camgomes@utad.pt (C.M.-G.); sandrinedsf@hotmail.com (S.S.F.); 2 Center for Research and Technology of Agro-Environmental and Biological Sciences (CITAB-UTAD), Quinta de Prados, 5001-801 Vila Real, Portugal; tatiana.andreani@fc.up.pt; 3 Department of Pharmaceutical Technology, Faculty of Pharmacy, University of Porto, Rua de Jorge Viterbo Ferreira, 228, 4050-313 Porto, Portugal; ebsouto@ff.up.pt; 4 UCIBIO/REQUIMTE, Faculty of Pharmacy, University of Porto, Rua de Jorge Viterbo Ferreira, 228, 4050-313 Porto, Portugal; 5 GreenUPorto—Sustainable Agrifood Production Research Centre and Department of Biology, Faculty of Sciences, University of Porto, Rua do Campo Alegre s/n, 4169-007 Porto, Portugal

**Keywords:** glyphosate, imidacloprid, imazalil, oxidative stress markers, apoptosis, flow cytometry, partition coefficient, toxicity prediction

## Abstract

In this work, three pesticides of different physicochemical properties: glyphosate (GLY, herbicide), imidacloprid (IMD, insecticide), and imazalil (IMZ, fungicide), were selected to assess their cytotoxicity against Caco-2 and HepG2 cells. Cell viability was assessed by the Alamar Blue assay, after 24 and 48 h exposure to different concentrations, and IC_50_ values were calculated. The mechanisms underlying toxicity, namely cellular reactive oxygen species (ROS), glutathione (GSH) content, lipid peroxidation, loss of mitochondrial membrane potential (MMP), and apoptosis/necrosis induction were assessed by flow cytometry. Cytotoxic profiles were further correlated with the molecular physicochemical parameters of pesticides, namely: water solubility, partition coefficient in an *n*-octanol/water (Log P_ow_) system, topological polar surface area (TPSA), the number of hydrogen-bonds (donor/acceptor), and rotatable bonds. In vitro outputs resulted in the following toxicity level: IMZ (Caco-2: IC_50_ = 253.5 ± 3.37 μM, and HepG2: IC_50_ = 94 ± 12 μM) > IMD (Caco-2: IC_50_ > 1 mM and HepG2: IC_50_ = 624 ± 24 μM) > GLY (IC_50_ >>1 mM, both cell lines), after 24 h treatment, being toxicity time-dependent (lower IC_50_ values at 48 h). Toxicity is explained by oxidative stress, as IMZ induced a higher intracellular ROS increase and lipid peroxidation, followed by IMD, while GLY did not change these markers. However, the three pesticides induced loss of MMP in HepG2 cells while in Caco-2 cells only IMZ produced significant MMP loss. Increased ROS and loss of MMP promoted apoptosis in Caco-2 cells subjected to IMZ, and in HepG2 cells exposed to IMD and IMZ, as assessed by Annexin-V/PI. The toxicity profile of pesticides is directly correlated with their Log P_ow_, as affinity for the lipophilic environment favours interaction with cell membranes governs, and is inversely correlated with their TPSA; however, membrane permeation is favoured by lower TPSA. IMZ presents the best molecular properties for membrane interaction and cell permeation, i.e., higher Log P_ow_, lower TPSA and lower hydrogen-bond (H-bond) donor/acceptor correlating with its higher toxicity. In conclusion, molecular physicochemical factors such as Log P_ow_, TPSA, and H-bond are likely to be directly correlated with pesticide-induced toxicity, thus they are key factors to potentially predict the toxicity of other compounds.

## 1. Introduction

Pesticides comprise a vast number of compounds, with different chemical structures, different organisms, and molecular targets as well as biological effects being used for crop and postharvest protection in the agricultural sector [1,2]. The term pesticide includes fungicides, herbicides, insecticides, molluscicides, nematicides, rodenticides, plant growth regulators, and other compounds which are classified according to the target pest [2,3]. For example, herbicides, fungicides, and insecticides are commonly used worldwide in agriculture to kill weeds or unwanted plants, fungi, and insects, respectively [3,4,5]. If, on the one hand, the use of pesticides has brought great benefits, both in increasing the availability and quality of food and for public health in general [6], on the other hand, exposure to them is a source of various diseases for animals and humans [2,7]. Given the various regulatory standards for the use of pesticides, it is estimated that they are found in trace amounts in food products, representing a low risk of toxicity for consumers [2,7,8]. However, for some pesticides, their molecular structure can make their elimination from the body difficult and therefore chronic exposure can be toxic, resulting in several diseases depending on the dose and exposure time, including metabolic toxicity, neurotoxicity, cancer, and endocrine disruption, among others [7].

Among the various biological effects of pesticides, their action on cellular oxidative stress has been highlighted by increasing the levels of reactive oxygen species (ROS) and/or reactive nitrogen species (RNS) that generate damage to DNA, proteins, and lipids [1,9,10,11]. For instance, paraquat (1,1′-dimethyl-4,4′-bipyridinium dichloride), a non-selective herbicide, at non-cytotoxic concentrations, induces oxidative DNA damage in HepG2 cells [12]. Imidacloprid, a neonicotinoid insecticide, induces oxidative stress and DNA damage on non-target organisms, namely various fish species such as *Danio rerio* (zebrafish) [13], *Prochilodus lineatus* [14], and *Oreochromis niloticus* (Nile tilapia) [15], affecting various organs (e.g., gills, kidney, brain, liver), as assessed by the levels of stress defence enzyme activity and by comet assay [13,14,15]. Imidacloprid was also reported to induce oxidative stress and inflammation in the hepatic and central nervous system of rats [16] and was associated with multiple neurobehavioral aberrations in adolescent and adult rats [17]. Moreover, using *Drosophila melanogaster*, imidacloprid at low doses promotes calcium influx into neurons triggering a fast increase in neuronal ROS level, affecting mitochondria, energy levels, and lipid environment, triggering metabolic and neurological impairments [18]. Oxidative stress and DNA damage was also observed among workers exposed to pesticides, as seen in assays of blood cells and by the presence of selected stress metabolites in urine [1]. Chronic exposure of mice to imazalil (a broad-spectrum fungicide) induces hepatotoxicity, showing disturbed hepatic metabolism and oxidative stress [19]. Thus, many studies have reported oxidative stress induced by pesticides, namely using aquatic models to mimic environmental exposure, animal models (such as rat and mouse), and *Drosophila*, among others, through chronic or acute exposure [18]. However, when using pesticide preparations, the toxic effect of the excipient compounds (e.g., emulsifiers and surfactants) can be higher than that of the pesticide, as recently revised for glyphosate (an herbicide) [4]. Heusinkveld and Westerink [20] studied the effect of various pesticides (dieldrin, dinoseb, imazalil, lindane, and rotenone) in four cell lines (PC12, SHSY5Y, MES23.5 and N27 cells), and reported the cytotoxic effect (concentrations up to 100 μM) dependent on the cell line and on the pesticide, also observed that ROS increase is not necessarily accompanied by mitochondrial activity changes, and vice versa. This is a very interesting issue, as pesticides may affect cells that are supposedly not targetable and also the effect on some cell lines might not be extrapolated to others. Thus, it is necessary to increase the knowledge about the effect of pesticides in different cell lines to assess the various toxicological effects, whether induced by environmental, occupational, or food exposure.

**Figure 1:**
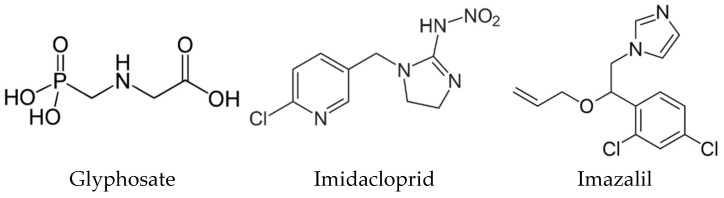
Chemical structure of glyphosate, imidacloprid, and imazalil.

To perform this study three pesticides: glyphosate (GLY), imidacloprid (IMD) and imazalil (IMZ), an herbicide, an insecticide, and a fungicide, respectively, were selected as they have different chemical structures (Figure 1), are directed to different molecular targets (at target pests), and have different physicochemical properties, such as water solubility and partition coefficient. *N*-phosphonomethyl-glycine (GLY) inhibits the 5-enolypyruvylshikimate-3-phosphate synthase (EPSPS; EC 2.5.1.19) metabolic pathway which is crucial for the biosynthesis of essential metabolites (e.g., the amino acids phenylalanine, tyrosine, or tryptophan); EPSPS is produced and present in plants, fungi, and some microorganisms, but not in animals [4,21,22]. IMD belongs to the neonicotinoid insecticide class, with a chemical structure similar to nicotine it is selective for nicotinic acetylcholine receptor (nAChR), showing higher affinity for insect nAChR than for vertebrate nAChR [23,24]. Although developed to target insects nAChR, adverse effects on vertebrate cell and in vivo animal models were reported, taken together with the fact that high levels of IMD and of its metabolites have been detected in several food products, such as honey, fruits, and vegetables [25]. IMZ (or enilconazole) is a broad spectrum systemic fungicide that blocks ergosterol biosynthesis by targeting cytochrome P450-dependent sterol 14α-demethylase (Cyp51; EC 1.14.13.70) and blocking the production of C14-demethylation of lanosterol, a precursor of ergosterol, [26]. IMZ is used worldwide to prevent postharvest decay of fruit (bananas, citrus fruit, and others), vegetables, and ornamentals [27].

Bearing in mind that pesticides target metabolic pathways that are not present in mammals, or have very low affinity for molecular targets in mammals (in the case of neonicotinoids), it is questionable whether the molecular nature of pesticides have a great influence on its toxicity and if the physicochemical properties inherent to the pesticide molecule rule its nonspecific toxicity.

Thus, the main aim of this study was to assess the toxicity of three pesticides, GLY, IMD, and IMZ, on human epithelial colorectal adenocarcinoma (Caco-2) and human hepatocellular carcinoma (HepG2) as models to mimic gastrointestinal and hepatic exposure, respectively, by assessing the cell viability and several oxidative stress markers (e.g., ROS and glutathione content) and apoptosis by flow cytometry. Moreover, we also aimed to compare the degree of induced oxidative stress and apoptosis with the pesticides’ main physicochemical parameters, namely the water solubility, the partition coefficient in the *n*-octanol/water (Log P_ow_) system, the topological polar surface area, and hydrogen-bonds, in order to extrapolate to other cell types and to predict effects of chronic exposure.

## 2. Results and Discussion

### 2.1. Assessment of Pesticides Effect on Caco-2 and HepG2 Cells Viability

**Figure 2:**
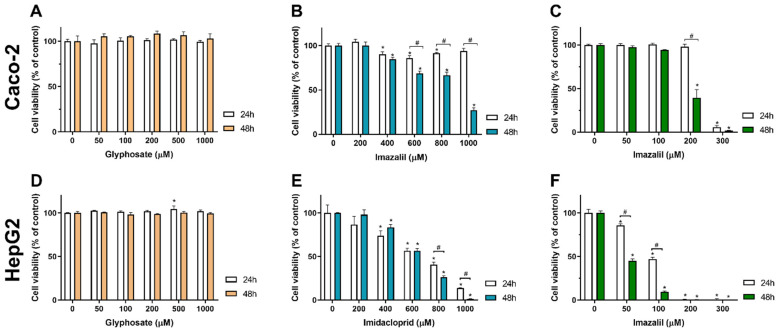
Effect of glyphosate (**A**,**D**), imidacloprid (**B**,**E**), and imazalil (**C**,**F**) on Caco-2 and HepG2 cells viability. Cells were exposed to different concentrations of the mentioned pesticides, for 24 h or 48 h, then cell viability was assessed by Alamar Blue (see methods for details). Data are presented as mean ± SD (*n* = 4) and as percentage of control (non-exposed cells). Statistical significance (*p* < 0.05) between sample and respective control is denoted by an *, and # (over a square bracket) denotes statistical significance for the same concentration at different time points.

In the present research we report the comparison of cytotoxicity, oxidative stress, and cell death induction by three pesticides, GLY, IMD, and IMZ, in Caco-2 and in HepG2 cells, which are cell models of intestinal abortive epithelium and of hepatocytes, respectively. Aiming to compare the effect of these pesticides, this study started by assessing the effect of the three pesticides on Caco-2 and HepG2 cell viability. Cells were exposed to a range of concentrations (up to 1 mM) of the pesticides for two periods of incubation (24 h and 48 h), then cell viability was assessed by the Alamar Blue assay (see methods for details), and results were calculated as percentage of control (non-exposed cells). Table 1 shows the pesticide concentration required to reduce cell viability in 50% (i.e., IC_50_ values) for Caco-2 and HepG2 cells subjected to the pesticides for 24 h and 48 h of exposure. The IC_50_ values were obtained from dose-response curves as that shown in Figure 2.

As observed from Table 1 and Figure 2, GLY was not cytotoxic to both cell lines at both incubation times, providing IC_50_ values above 1 mM. No significant decrease in cell viability was observed in cells exposed to GLY at concentrations up to 1000 μM, for both exposure times, as illustrated in Figure 2A,D, for Caco-2 and HepG2 cells, respectively. On the other hand, IMD produced a dose-dependent toxicity in Caco-2 and HepG2 cells (Table 1, Figure 2B,E). Nevertheless, the hepatocyte cell model presents higher sensitivity to IMD exposure, presenting lower IC_50_ values, for both 24 h and 48 h of exposure, compared with Caco-2 cells (Table 1). IMZ was the most cytotoxic pesticide, and induced dose- and time-dependent toxicity producing the lower IC_50_ values for both cell lines, at both exposure times. As observed in Table 1, for Caco-2 the IMZ IC_50_ at 24 h is 1.35-fold higher than the IC_50_ observed at 48 h of exposure, while in HepG2 a 2-fold decrease in the IC_50_ was obtained when doubling the exposure time. Comparing between cell lines, the IMZ IC_50_ values for Caco-2 cells are 2.7-fold and 4-fold higher than those of HepG2 cells, at 24 and 48 h of exposure, denoting the higher sensitivity of HepG2 cells to this pesticide, and at the same time a higher tolerance of intestinal cells to this pesticide.

Other studies have also shown that glyphosate per se has low toxicity in in vitro cell models; however, when using commercially available glyphosate formulations (e.g., RoundUp^®^) the toxicity is much higher which is attributed to the other compounds present in the formulation, as recently reviewed by Martins-Gomes, et al. [4]. Using the GLY standard, concentrations higher than 10 mg/mL (~59 mM) disrupted Caco-2 monolayers and induced loss of membrane integrity [28]. However, this concentration is 59-fold higher than the highest here tested, we aimed to test concentrations closer to the real exposure doses and to use comparable concentrations between pesticides. Using occupational concentrations of GLY, Kašuba, et al. [29] reported no reduction in HepG2 cell viability. However, concentrations higher than 3 mM were reported as genotoxic to HepG2 cells and to induce oxidative stress [30].

Regarding the effect of IMD on HepG2, Guimarães, et al. [31] reported dose-dependent reduction in cell viability for the range 0.5–2.0 mM and 0.25–2.0 mM, at 24 h and 48 h of exposure, respectively. Values that corroborate with the IMD toxicity against HepG2 cells can be seen in Table 1. For Caco-2, slightly different IC_50_ values have been reported; however, in different experimental conditions, for example in the study by Nedzvetsky, et al. [32], it was reported that Caco-2 cells exposed to concentrations higher than 0.25 µg/mL (~0.98 μM) for 96 h showed a reduction in cell viability (81% of cell viability 0.25 µg/mL) which is a value much lower than ours. However, these authors used a IMD formulation from Bayer, in which other compounds (e.g., emulsifiers and surfactants) might be the source of toxicity. In this study the IMD standard was used. IMD reduced the viability of L-929 cells (fibroblast cell line), as assessed by lactate dehydrogenase (LDH) release, with 50% LDH release increase at 500 μM [33], values that reflect the IC_50_ values were obtained (Table 1). 

Concerning IMZ toxicity against cell lines, there are few studies being limited to Caco-2 cells. Tao, et al. [34] reported, for Caco-2 cells exposed to IMZ for 24 h, an IC_50_ of 30 µg/mL (~100 µM), which is 2.53-fold lower than the IC_50_ here reported (Table 1); however, while in our study Caco-2 cells were seeded at 5 × 10^4^ cells/mL (100 μL/well) and allowed to adhere for 48 h, Tao, et al. [34] only allowed 24 h which also implies lower cell number when tested. Concerning the effect of IMZ on HepG2 cells, to the best of our knowledge, this is the first work reporting it.

Thus, having the cytotoxic profile of these pesticides in Caco-2 and HepG2 cells, we aimed to assess their effect on oxidative stress and apoptosis using flow cytometry. The intracellular reactive oxygen species (ROS), intracellular content in glutathione (GSH), and lipid peroxidation and mitochondrial membrane potential (MMP) were measured as markers of oxidative stress, and the inversion of phosphatidylserine and/or disruption of cell membrane, as markers of apoptosis and/or necrosis, in Caco-2 and HepG2 cells exposed to the pesticides for 24 h.

For the flow cytometry study, according to data in Table 1, three concentrations of each pesticide were chosen, aiming to compare the effects between cell lines and between pesticides. As these are adherent cells that need trypsin treatment before cytometry analysis, in order to avoid great loss of cells and high amount of debris which make the analytical procedures difficult, in some cases values just below IC_50_ were chosen (see Table 2). 

### 2.2. Evaluation of Oxidative Stress Markers

**Figure 3:**
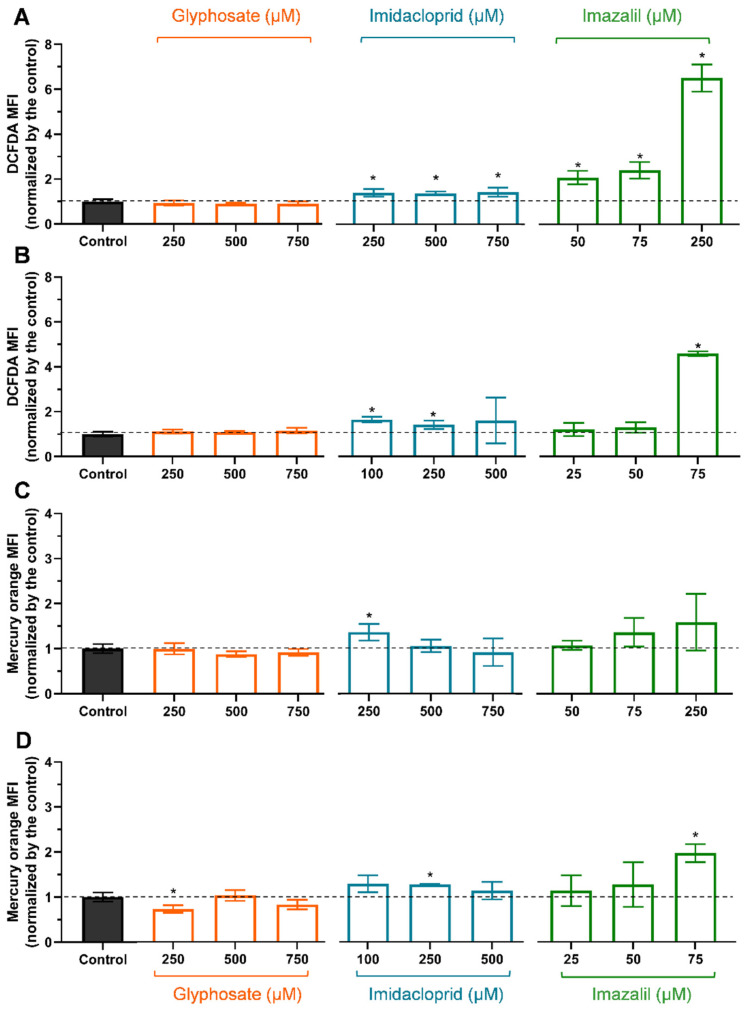
Changes in intracellular reactive oxygen species ((**A**): Caco-2; (**B**): HepG2) and glutathione ((**C**): Caco-2; (**D**): HepG2) contents, induced by cells exposure to GLY, IMD, and IMZ, for 24 h, at the indicated concentrations. Results are presented as mean ± SD. Values were normalized to control cells (non-exposed cells). Significant statistical differences between the control and samples are denoted as “*” when *p* < 0.05.

As the available literature often reports pesticides in general as oxidative stress inducers, mainly through in vivo studies using fish models to mimic environmental exposure (e.g., [13,14]), we aimed to evaluate and compare the mechanisms behind the toxicity observed (Table 1 and Figure 2), for GLY, IMD, and IMZ, at the oxidative stress level. Thus, flow cytometry analysis of oxidative stress markers was performed in Caco-2 and in HepG2 cells were exposed for 24 h to the selected concentrations of respective pesticides (see Table 2). Although different toxicity profiles were observed, the pesticides’ concentrations were chosen so that the concentration values allowed comparison between cell lines and whenever possible between pesticides. Figure 3 presents the changes in ROS and GSH content, in Caco-2 and HepG2 cells exposed to the pesticides, for 24 h, at three different concentrations. 

According to the results obtained for cell viability assays, both cell lines exposed to concentrations up to 750 μM of GLY presented control-like ROS levels (Figure 3A,B), further supporting the absence of toxicity at least at the oxidative level. We did not decide to use higher glyphosate concentrations because we needed to compare between pesticides, and IMD and IMZ could not be applied at higher concentrations as they are more toxic (Table 1). Moreover, according to the literature, the highest values of glyphosate food products were reported for soybean reaching 8800 μg/kg [35,36] (i.e., 50 μmol/kg), thus the values used in this study are above the exposure levels, since glyphosate has high water solubility being eliminated in urine.

IMD induced a significant (*p* < 0.05) increase in ROS content, in both cell lines, at the lowest tested concentration (Caco-2: 250 μM and HepG2: 100 μM). The increase in ROS content was more pronounced in HepG2 cells than in Caco-2 cells, as 750 μM of IMD increased Caco-2 cells ROS content in 1.42-fold compared with the control, while HepG2 cells exposed to 500 μM showed a 1.59-fold increase. This also explains the higher sensitivity of HepG2 cells to IMD compared with Caco-2 cells. The effect of IMD on ROS content was recently reported by Guimarães, et al. [31], indicating that IMD at 500 μM doubled ROS content in HepG2 cells after 24 h of incubation, a value that is only slightly higher that that here reported. Moreover, to the best of our knowledge this is the first report on the IMD-induced ROS increase in Caco-2 cells.

Concerning IMZ, its high toxicity (Table 1) directly correlates with its high ability to induce oxidative stress in both cell lines (Figure 3). At the highest tested concentration, Caco-2 cells (IMZ: 250 μM) produced a 6.5-fold increase in ROS content while HepG2 cells exposed at 75 μM presented a 4.56-fold increase in ROS content. However, comparing both cell lines exposed to 75 μM IMZ, it is evident that the ROS content in HepG2 is about twice than that in Caco-2 cells (at 75 μM IMZ, ROS content increased 2.40-fold; Figure 3A). Thus, both IMD and IMZ induce increased oxidative stress events in HepG2 compared with the same concentration of pesticides in Caco-2 cells. Using fluorescence microscopy, Tao, et al. [34] reported an increase in ROS content in Caco-2 cells exposed to IMZ at 30 µg/mL (~100 µM), which corroborates our data. To the best of our knowledge this is the first study reporting the effect of IMZ on HepG2 ROS content.

In response to oxidative stress, cells activate antioxidant defences that include increasing the levels of GSH. As observed in Figure 3C, Caco-2 cells exposed to GLY did not show GSH levels different from control cells (*p* > 0.05), despite the slight decrease on average GSH levels, as observed for ROS content (Figure 3A). However, the initial decrease in GSH content of HepG2 cells induced by GLY (Figure 1D) is statistically significant from control cells, but no statistical significance is observed at the higher concentrations. 

Caco-2 (Figure 3C) and HepG2 (Figure 2D) increased GSH levels in response to IMD exposure, but as IMD concentration induces an increase in ROS content a depletion in GSH is observed. As observed, IMD induced an increase in the mean values of GSH content at all tested concentrations, most likely in response to the increase in ROS content. 

In the case of cells exposed to IMZ, which present the higher increase in ROS, both cell lines show a dose-dependent increase in GSH content in response to the dose-dependent increase in ROS (Figure 3). On average, Caco-2 increased GSH content by 1.59-fold at the highest IMZ concentration (250 μM) while HepG2 exposed to 75 μM IMZ increased GSH by 1.97-fold. This might denote the intrinsic metabolism of HepG2 cells that have a high capacity to generate and regenerate GSH in response to oxidative stress [37]. However, other mechanism might explain the higher sensitivity of these cells to IMZ. 

The presence of high levels of ROS attaches cell components, such as DNA, proteins, and lipids. Membrane lipids, namely those containing carbon–carbon double bonds, are susceptible to oxidation in a three step process: initiation, propagation, and termination [38,39]; once initiated, a propagation of chain reaction occurs until termination products are produced [38], among the various products malondialdehyde (MDA) production has been extensively studied using several methods. Using the fluorescence probe DHPE-FITC and flow cytometry it is possible to evaluate the degree of lipid peroxidation in cells exposed to oxidative stress [40,41]. Under oxidative stress, a decrease in MFI as a consequence of the fluorescein moiety of DHPE oxidation due to the activity of lipid peroxidation products is observed [40,41,42]. 

**Figure 4:**
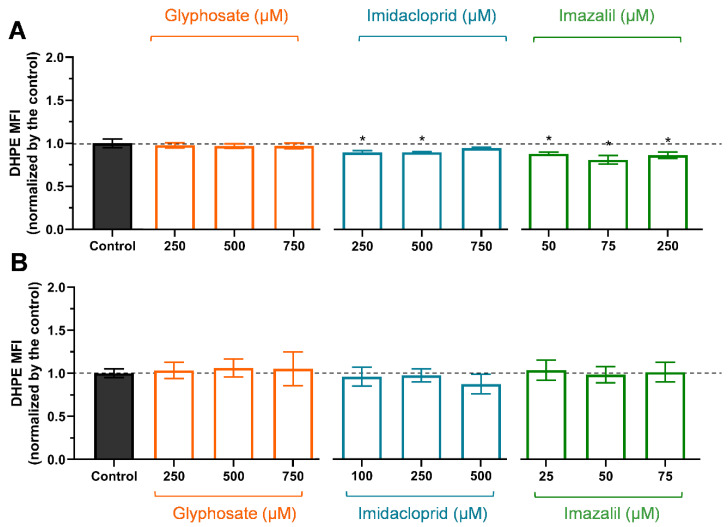
Assessment of lipid peroxidation in Caco-2 (**A**) and in HepG2 (**B**) cells exposed to GLY, IMD, and IMZ, for 24 h, using flow cytometry and DHPE-FITC probe. Results were normalized to control (non-exposed cells) and are presented as mean ± SD (*n* = 3 independent experiments). Significant statistical differences between the control and samples are denoted by “*” when *p* < 0.05.

Results obtained for lipid peroxidation (LP), induced by GLY, IMD, and IMZ, in Caco-2 and HepG2 cells are presented in Figure 4. When LP occurs DHPE-FITC interacting with lipid peroxidation products leads to a loss of the fluorescence moiety, thus LP is observed as a decay in the MFI.

As observed, GLY did not produce significant LP in Caco-2 (Figure 4A) or in HepG2 (Figure 4B) cells (*p* > 0.05), corroborating with control-like ROS content (Figure 3). In Caco-2 cells (Figure 4A), IMD and IMZ induced significant LP, with IMZ-induced LP being more pronounced (*p* < 0.05, to all concentrations), corroborating with the production of higher levels of ROS (Figure 3A). In HepG2 cells (Figure 4B), 750 μM IMD, on average, increased the LP; however, the values are not statistically significant (*p* > 0.05), comparing to control. In HepG2 cells, IMZ concentrations up to 75 μM did not increase lipid peroxidation (*p* > 0.05). The observation that the IMZ-induced LP is higher in Caco-2 than in HepG2 cells can be explained by ROS and GSH contents (Figure 3). Although Caco-2 cells are less sensitive to IMZ (Table 1), we observed higher ROS levels in Caco-2 exposed to IMZ at IC_50_ (~250 μM) than in HepG2 at IC_50_ (~75 μM), but at these IMZ concentrations HepG2 presents higher GSH content than Caco-2 cells (Figure 3). We assumed that the higher GSH levels in HepG2 cells eliminated ROS and prevented LP. Comparing both cell lines exposed to 75 μM IMZ, we clearly observe that oxidative markers in Caco-2 cells are at lower levels than in HepG2 cells, although hepatocytes are specialized in xenobiotic metabolism [37,43]. However, enterocytes forming the intestinal barrier play an important role in preventing ingested xenobiotics from entering the blood flow; these cells present a vast number of efflux transporters on the apical membrane, such us multidrug resistance proteins (MDR, MDR1), ATP-binding cassette proteins (ABC-transporters), and others [44]; they are specialized in the efflux of xenobiotics such as pesticides, and therefore are likely more capable of countering pesticide-induced toxicity. Thus, we may infer that enterocytes are specialized in avoiding xenobiotics.

### 2.3. Pesticide-Induced Mitochondria Membrane Depolarization

Given the role of mitochondria in both oxidative stress and cell death (apoptosis), we evaluated the mitochondrial membrane potential (MMP) using the JC-1 probe (see methods for details). This probe accumulates in the mitochondria in the form of J-aggregates when in normal mitochondrial conditions (polarized) and presents red fluorescence. By contrast, in unhealthy cells, when depolarization occurs, JC-1 enters the mitochondria in lower concentrations forming monomers inducing a fluorescence shift to green fluorescence, thus the loss of MMP is observed through flow cytometry as a reduction in red (FL2) and an increase in green (FL1) fluorescence, or as a reduction in red/green (FL2/FL1) fluorescence ratio [45,46].

**Figure 5:**
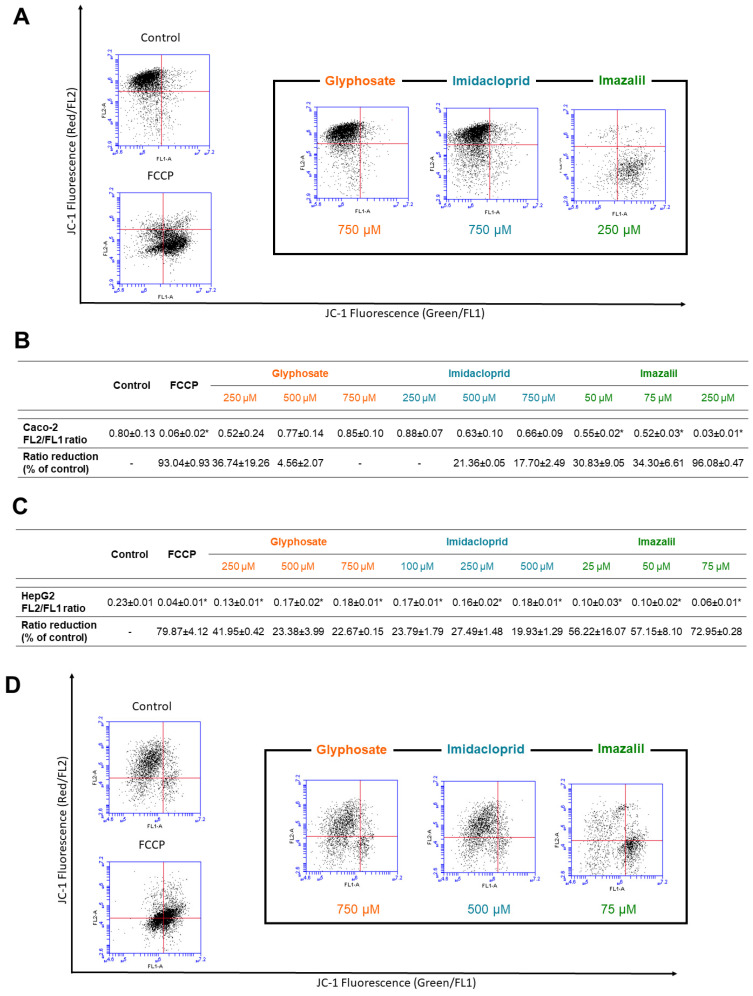
Assessment of changes on mitochondrial membrane potential (MMP) in Caco-2 (**A**,**B**) and HepG2 (**C**,**D**) cells exposed to GLY, IMD, and IMZ for 24 h, at indicated concentrations. Flow cytometry dot plots of Caco-2 (**A**) and HepG2 (**D**) cells exposed to the highest concentrations of each pesticide. The calculated FL2/FL1 ratio for Caco-2 (**B**) and HepG2 (**C**) cells exposed to 3 different concentrations of each pesticide, and the percentage of FL2/FL1 rate decay comparing to respective control. Results are presented as mean ± SD. Significant statistical differences between the control and samples are denoted as “*” when *p* < 0.05.

The results of pesticides-induced changes on MMP are presented in Figure 5.

Figure 5A shows the scatter plots obtained from flow cytometry analysis of MMP in Caco-2 cells. Control viable cells cluster in the upper-left quadrant, due to staining with JC-1 aggregates red fluorescence (FL2) and no green fluorescence (FL1). On the other hand, when cells are treated with carbonyl cyanide *p*-trifluoromethoxyphenylhydrazone, (FCCP) a protonophore, dues to cells loss of MMP and fluorescence shift to green fluorescence, an increase in FL1 is observed, and thus viable cells cluster toward the lower-right quadrant. FCCP was used as positive control. Regarding Caco-2 cells subjected to the pesticides (Figure 5A,B); GLY did not induce significant changes on MMP, as seen by the control-like dispersion in the scatter plot for GLY at 750 μM (Figure 5A) and by the calculated % of rate decay in FL2/FL1 (Figure 5B). None of GLY concentrations induced significant MMP changes (*p* > 0.05), as seen in Figure 5B. However, as observed, IMZ induced high changes in Caco-2 MMP, with a scatter plot similar to that of FCCP (Figure 5A), with a major cluster in the lower-right quadrant indicating high mitochondrial membrane depolarization, and thus correlating with the results obtained for ROS content (Figure 3A). We may infer that elevated ROS induces loss of MMP in Caco-2 cells. In general, Caco-2 cells subjected to IMD did not show significant loss of MMP (*p* > 0.05, Figure 5B).

Regarding HepG2 cells (Figure 5C,D), all pesticides induced significant reduction in MMP at all tested concentrations. Nevertheless, as observed for Caco-2 cells, IMZ was the most toxic pesticide, with higher impact in HepG2 cells, where at 75 μM the FL2/FL1 ratio was reduced 3.38 times (~73% of reduction, in relation to the negative control), while the same concentration in Caco-2 cells reduced the FL2/FL1 ratio by 1.54 times (i.e., ~34% reduction in relation to respective control). 

### 2.4. Evaluation of Pesticide-Induced Apoptosis/Necrosis

**Figure 6:**
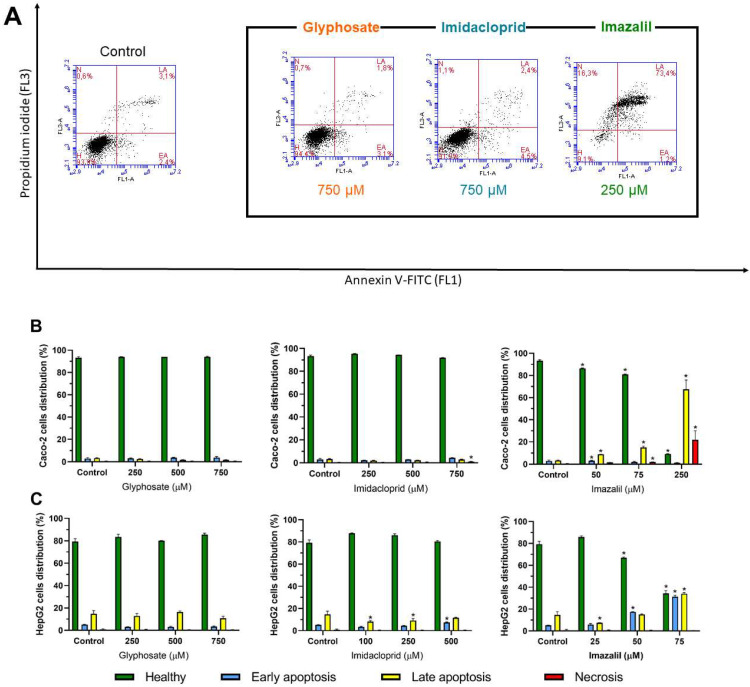
Evaluation of pesticide-induced apoptosis/necrosis in Caco-2 and HepG2 exposed to GLY, IMD and IMZ, for 24 h. (**A**): Representative flow cytometry scatter plots obtained from double-stained Caco-2 cells exposed for 24 h to the highest concentration of each pesticide (as denoted). (**B**) and (**C**): Graphical representation of Caco-2 (**B**) and HepG2 (**C**) cells distribution by the 4 main populations (H, healthy; EA, early apoptosis; LA, late apoptosis; N, necrosis). Results were obtained after analysis of dot plots as represented in A. Results are presented as mean ± SD (*n* = 3). Significant statistical differences between the control and samples are denoted as “*” when *p* < 0.05.

Due to the loss in cell viability and to the increase in oxidative stress as accompanied by the loss in MMP, which are signs of apoptosis induction, we further analysed the effect of the three pesticides, at selected concentrations, on apoptosis/necrosis induction by Annexin-V-FITC/PI double staining (see methods for details). Figure 6 shows the obtained results for Caco-2 (Figure 6A,B) and for HepG2 (Figure 6C) cells.

As observed in Figure 6A (left panel), control Caco-2 cells are grouped in the lower-left quadrant of the dot plot indicating negative staining to Annexin-V and to PI. Exposure to GLY did not produce significant changes in the % of cell distribution through the various populations (Figure 6B). The identical pattern was observed for Caco-2 cells exposed to IMD. Contrarily, IMZ induced a dose-dependent increase in late apoptosis (Figure 6B, rightmost panel, yellow bars) and at the highest concentration ~23% of cells were in necrosis, as compromised cell membranes lead to the entrance of PI.

Concerning HepG2 cells (Figure 6C), as expected, GLY did not induce significant changes on cell population distribution (comparing to control), but IMD at the highest concentration increased the cell population in late apoptosis (*p* < 0.05). IMZ, at the tested concentrations dose-dependently increased apoptosis, in particular late apoptosis (Figure 6C, rightmost panel, yellow bars). However, at the highest tested concentration (~IC_50_, 75 μM) there was no necrotic events recorded contrarily to that observed for Caco-2 subjected to IMZ at concentration ~IC_50_, (250 μM), this can be explained by the fact that the IC50 concentration induces higher loss of MMP in Caco-2 cells than in HepG2 cells, indicating the inability to produce ATP and thus inducing cell death. To the best of our knowledge this is the first study comparing the effect of three different pesticides concerning the effect of MMP loss using JC-1, which is much more accurate than any other probe due to organelle distribution and spectral characteristics [46] with apoptosis/necrosis induction. 

As the chosen pesticides have different molecular structures and characteristics, we further analysed the correlation of pesticides toxicity with the molecular characteristics of the pesticides by performing a comparison that is identical to the targetability and toxicity prediction or drugs used in oral route. This may explain some differences in toxicity, namely between pesticides, as none of the used cell lines are target for them.

### 2.5. Comparing the Cytotoxic Profile of Pesticides with Their Physicochemical Characteristics

As mentioned, these cells lines do not have relevant molecular targets to the tested pesticides. For both cell lines, the toxicity rank is GLY < IMD < IMZ, implying that intrinsic molecular characteristics of pesticides may be subjacent to toxicity.

In Table 3 we present the most relevant physicochemical properties of GLY, IMD, and IMZ which we consider relevant for toxicity. 

The toxicity of xenobiotics (as pesticides) is highly dependent on defined characteristics which facilitate the xenobiotic–cell membranes interaction as well as its ability to permeate the cell. In pharmaceutical development of drugs, several rules have been described and applied aiming to predict drug targetability to the cell, which may provide insights about pesticides-induced toxicity. Lipinski, et al. [51] described a “rule of 5”, in which key molecular physicochemical properties are likely to induce poor oral absorption or permeation: MW > 500, Log P_ow_ > 5, and hydrogen-bond (H-bond) donors and acceptors > 5 and >10, respectively. Moreover, cellular molecules, such as biological transporters with affinity for these drugs, are exceptions of those rules [51]. Later, Lipinski [52] and Veber, et al. [53] updated the rule, and added the rotatable bonds count (>10), which is linked to higher permeability through barriers (e.g., intestinal barrier), since an increased number in rotatable bounds decreases ligand affinity [52]. The polar surface area (PSA; ideally < 140 Å^2^) effect and the affinity for cellular xenobiotic efflux pumps were also considered. As reported, PSA directly correlates with drug/xenobiotic permeation, with higher significance than lipophilicity [52,53]. Other parameters, such as the quantitative estimate of drug-likeness and the quantitative structure-activity relationships (QSAR), provided a more accurate classification of the drug potential, which is based on the interactions of various criteria instead of an exclusive set of rules [54,55,56]. Although, these rules were intended for the characterization of drugs for oral intake drugs, not to be considered for other administration routes; these rules may thus also apply to oral exposure of pesticides. Considering the toxicity profile of GLY, IMD, and IMZ on Caco-2 and HepG2 cells, we made a correlation between the analysed parameters with the pesticides’ physicochemical characteristics, attempting to evaluate if the permeation and bioaccumulation potential of pesticides correlates with pesticides-induced toxicity in these cell models.

**Figure 7:**
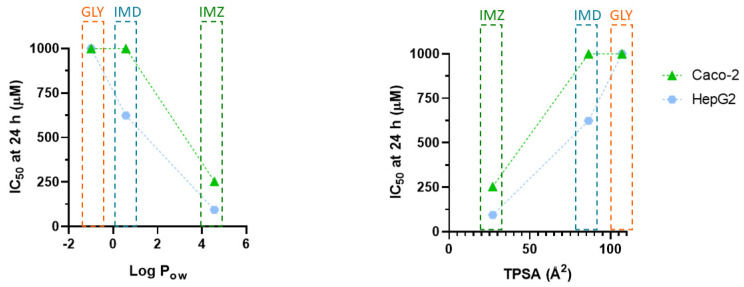
Correlation between pesticides-induced toxicity, represented by IC_50_ values, and pesticide Log P_ow_ (left panel) or with pesticide TPSA (right panel). The IC_50_ values were obtained from in vitro cytotoxicity assays (see Table 1) in cells subjected for 24 h to different concentrations of respective pesticide (see methods for details). Caco-2 and HepG2 IC_50_ values are denoted by green triangles and blue circles, respectively.

Concerning molecular weight (MW), all pesticides have MW < 500 (Table 3); however, toxicity (Table 1) is inversely proportional to MW. In relation to barrier permeation, xenobiotics may enter the cell passively (moving down the concentration gradient) or actively. In the first case, lipophilicity is critical in determining the ability to cross or be retained in the lipid bilayer. The partition coefficient in *n*-octanol/water system (Log P_ow_) is a common parameter to assess hydrophilicity or lipophilicity of a substance [57,58]. Several works determining absorption of drug using Caco-2 monolayer model and formulated with QSAR, have provided a positive correlation and usefulness of using Log P_ow_ to assess absorption of compounds. The average value of the optimum Log P_ow_ was 2.94 [56]. Concerning GLY, IMD, and IMZ, the reported Log P_ow_ values are: −1.0, 0.57, and 4.56, respectively (Table 3). As seen, IMZ has the highest Log P_ow_ which is also close to the ideal average value (2.94) reported by Hansch, et al. [56]. Thus, GLY has a negative Log P_ow_, is hydrophilic, and has high water solubility (Table 3) which is 8.57-fold and 20-fold higher than IMZ and IMD water solubility, respectively. Moreover, GLY has high polarity and very low solubility in organic solvents [59]. Considering the affinity for water (culture media) and to lipophilic phase (cell membranes), GLY has higher affinity for the aqueous phase, while IMD and IMZ present higher affinity for the lipophilic phase. Taking into account the toxicity data present in Table 1, the Log P_ow_ correlates directly with the observed toxicity, as IMZ shows the lowest IC_50_ values and the higher P_ow_ (Table 1 and Figure 7).

Topological polar surface area (TPSA) represents the sum of oxygen, nitrogen, and their linked hydrogen atoms surface area being the main functional groups, and it is also considered a relevant physicochemical property for molecules absorption predicting. Recent studies, using TPSA index, have proposed that molecules with TPSA > 140 Å^2^ have low membrane permeation, while those with TPSA < 60 Å^2^ have a higher ability to permeate biological membranes [60,61]. As observed in Figure 7, TPSA perfectly aligns with the observed toxicity. GLY has the highest TPSA value (107 Å^2^), while IMD (86.3 Å^2^) and IMZ (27 Å^2^) present progressively lower TPSA values and lower IC_50_ values (Figure 7; Table 1). These data show that the physicochemical properties of molecules that favour cell membrane interaction and/or permeation are predictors of cell toxicity.

**Figure 8:**
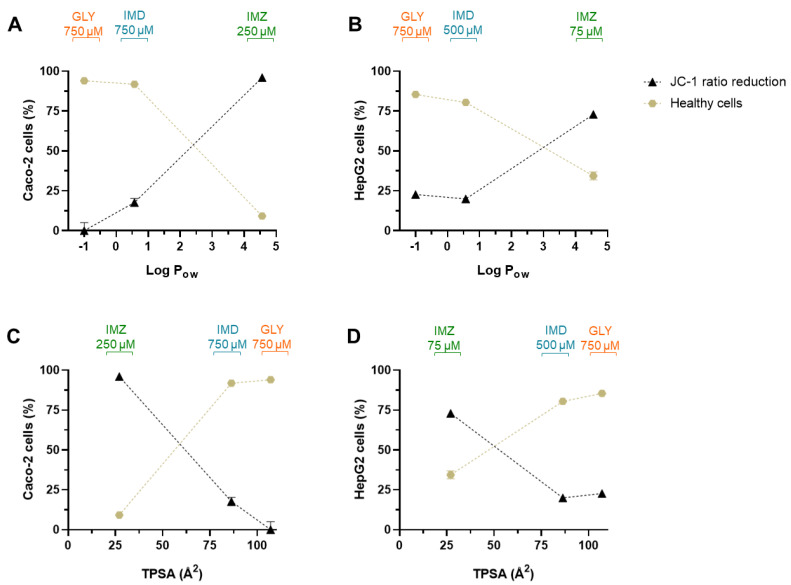
Correlation between loss of MMP (assessed as % in FL2/FL1 ratio reduction, in relation to negative control cells; obtained from Figure 5) and pesticides Log P_ow_ (**A**,**B**) or with pesticides TPSA (**C**,**D**), for Caco-2 and HepG2 cells, as denoted. Moreover, correlations between the % of healthy cells (assessed in Annexin-V/PI assay, Figure 6) and pesticides Log P_ow_ (**A**,**B**) and with pesticides TPSA (**C**,**D**), for Caco-2 and HepG2 cells as denoted. Loss of MMP and % of healthy cells are denoted by black triangles and brown circles, respectively.

We also correlated the loss in MMP as the percentage of the JC-1 fluorescence ration decrease, and the percentage of healthy cells (which is inverse to apoptosis and necrosis) with pesticides Log P_ow_ and with pesticides TPSA, the results are presented in Figure 8.

As observed in Figure 8, pesticides Log P_ow_ directly correlates with the loss of MMP. Compounds with higher Log P_ow_ have higher affinity for the cell membranes, including membranes of intracellular organelles, disrupting membrane architecture/structure, and thus, in the case of mitochondria, the loss of integrity leads to loss of MMP and consequently reduces or abolishes the ability to synthesize ATP, leading to cell death. In fact, as also shown in Figure 8, the number of viable cells decays as loss in MMP augments, and both depend on Log P_ow_ increase.

From Table 1 and Figure 7, it is evident that Caco-2 cells are less sensitive to pesticides-induced toxicity than HepG2 cells. Thus, although there is a cell type dependent toxicity, the order of pesticides toxicity is the same, being in both cells the toxicity rank GLY < IMD < IMZ. It has been reported that compounds with TPSA < 60 Å^2^ are non-P-gp substrate molecules [62]. Thus, when correlating Log P_ow_ and TPSA values, molecules presenting low Log P_ow_ simultaneously with high TPSA tend to be more easily removed and be less toxic (e.g., GLY), while the opposite ratio (high Log P_ow_/low TPSA) increases the molecule toxicity [63] (e.g., IMZ). Even more, the role of multidrug resistance-associated protein 1 (MRP1) as a xenobiotic efflux pump is linked to TPSA. It has been proposed that molecules with higher TPSA are substrates of MRP1, and that conjugation with glutathione (GSH), as conjugation of GSH and xenobiotic is a standard mechanism to increase their efflux, increasing molecule’s TPSA, and thus increasing its transport, compared with its unconjugated form with lower TPSA [61,64], this may explain the reason why Caco-2 cells present lower levels of GSH but higher tolerability to the pesticides, compared with HepG2 at the same concentrations (Figure 3 and Figure 7, Table 3). As mentioned above, Caco-2 cells represent one model of the barriers between the exterior and the interior of the body; thus these cells are equipped with various types of efflux transporters, such as the P-glycoprotein (P-gp), MRPs, and others [65], aiming to prevent the absorption of xenobiotics through their extrusion. 

**Figure 9:**
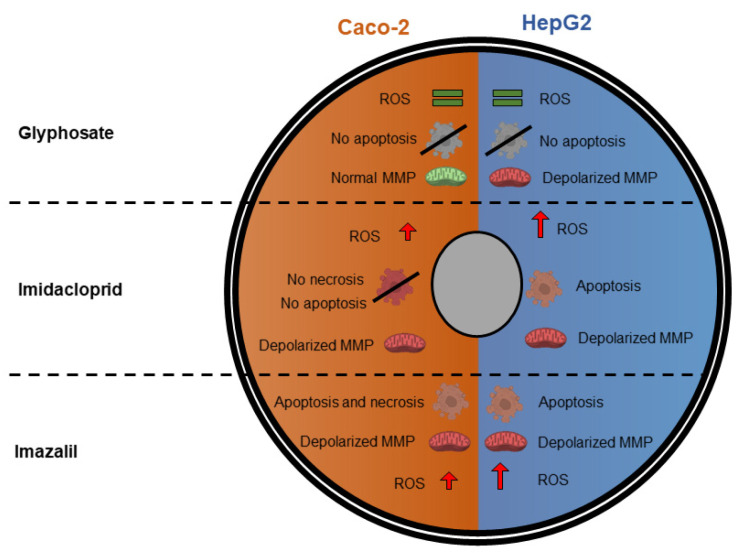
Summary of GLY-, IMD-, and IMZ-induced changes on oxidative stress and apoptosis/necrosis in Caco-2 and HepG2 cells.

Figure 9 schematically shows the effect of GLY, IMD, and IMZ on Caco-2 and HepG2 cells focusing the main markers of oxidative stress and occurrence, or not, of apoptosis and/or necrosis.

## 3. Materials and Methods

### 3.1. Materials and Reagents

Mercury Orange (1-(4-chloromercuriophenylazo)-2-naphthol), Annexin V-FITC, DCFDA (2′,7′-dichlorofuorescein diacetate), JC-1 (1H-Benzimidazolium, 5,6-dichloro-2-[3-(5,6-dichloro-1,3-diethyl-1,3-dihydro-2H-benzimidazol-2-ylidene)-1-propenyl]-1,3-diethyl-, iodide), propidium iodide (PI), glyphosate (PESTANAL^®^, analytical standard), imidacloprid (PESTANAL^®^, analytical standard), and imazalil (PESTANAL^®^, analytical standard) were obtained from Sigma-Aldrich (Merck, Germany). Versene, trypsin-EDTA, Dulbecco’s Modified Eagle Medium (DMEM), streptomycin, penicillin, L-glutamine sodium pyruvate, foetal bovine serum (FBS), and DHPE-FITC (*N*-(fluorescein-5-thiocarbamoyl)-1,2-dihexadecanoyl-*sn*-glycero-phosphoethanolamine) were obtained from Gibco (Alfagene, Invitrogen, Portugal). Alamar Blue^®^ was purchased from Invitrogen, Life-Technologies (Porto, Portugal). Caco-2 and HepG2 cell lines were purchased from Cell Lines Service (CLS; Eppelheim, Germany).

### 3.2. Evaluation of Pesticides Toxicity in Caco-2 and HepG2-Cells

#### 3.2.1. Cell Maintenance and Handling

In this study, to assess the cytotoxic activity of GLY, IMD, and IMZ, human epithelial colorectal adenocarcinoma (Caco-2), cell lines service (CLS), (Eppelheim, Germany) and human hepatocellular carcinoma (HepG2) (ATCC, Rockville, Maryland, USA) cells were used. Cells were cultured in complete culture media (Dulbecco’s Modified Eagle Media (DMEM), supplemented with 1 mM L-glutamine, 10% (*v*/*v*) foetal bovine serum (FBS), and antibiotics (penicillin at 100 U/mL, and streptomycin at 100 μg/mL)) and maintained at 5% CO_2_/95% air; 37 °C, controlled humidity, in an incubator. Cells were grown in T25 culture flasks to near confluence. Then, these adherent cells were treated with trypsin (trypsin-EDTA), for detachment and subculture, which was stopped using complete culture medium (1:1, trypsin:culture media) as soon as cells were detached (about 6 and 8 minutes of trypsin treatment for HepG2 and Caco-2 cells, respectively). Detached cells were gently re-suspended with a Pasteur pipette and then a sample was counted using an automated cell counter (TC10™, BIORAD, Portugal). A suspension of cells in fresh culture media, at 5 × 10^4^ cells/mL was prepared to seed into 96-well microplates (100 µL/well). After seeding, cells were placed in the incubator for 48 h (to adhere and stabilize) before being used, for other details see [66,67]. 

#### 3.2.2. Cell Viability/Cytotoxicity Assay

Cell viability was assessed using the Alamar Blue^®^ (AB) assay, after exposure to the pesticides. In summary, cells seeded into 96-well microplates were subjected to various concentrations of the pesticides (GLY: 0–1 mM; IMD: 0–1 mM; IMZ: 0–0.3 mM). To prepare the test solutions, the appropriate volume of the pesticide stock solution was diluted in FBS-free culture media just before its application to the cells. Stock solution of GLY was prepared in water (at 40 mM), and stock solutions of IMD and IMZ were prepared in DMSO (at 20 mM). After 24 h or 48 h of exposure, the test solutions were removed and the Alamar Blue solution (100 µL/well; 10% (*v/v*) in FBS-free medium) was immediately added to the cells, followed by a further 5 h incubation. The absorbance was then read at 570 and 620 nm using a microplate reader (Multiskan EX; MTX Lab Systems, Inc., Bradenton, FL, USA) and the percentage of the AB reduction was calculated according to the manufacturer’s instructions, as described before [66]. Cell viability, at each condition, was calculated and expressed as percentage of control cells (non-exposed cells) [66].

The concentrations needed to reduce cell viability by 50% (IC_50_) were calculated from three independent experiments (each one performed in quadruplicates), as described [68].

### 3.3. Flow Cytometry Assays for Oxidative Stress, Mitochondrial Membrane Depolarization and Cell-Death Evaluation

Pesticide-induced oxidative stress and apoptosis/necrosis events were analysed by flow cytometry. For this, one colour and two-colour flow cytometry assays were performed, using a BD Accuri™ C6 cytometer (Franklin Lakes, NJ, USA) equipment for data acquisition. Each assay was performed at least in triplicate (n = 3) and 5000 gated events were collected from each sample. Data analysis was performed using BD Accuri™ C6 Software, version 1.0.264.21 supplied by Becton Dickinson (Franklin Lakes, NJ, USA).

#### 3.3.1. Cell Handling for Flow Cytometry Assays

Caco-2 and HepG2 cells (handled as described in Section 3.2.1) were seeded in 12-well microplates (at 5 × 10^4^ cells/mL density; 750 µL/well), and allowed to adhere and stabilize for 48 h in the incubator. According to data obtained from cell viability results, cells were exposed to GLY (Caco-2 and HepG2: 250, 500, 750 µM), IMD (Caco-2: 250, 500, 750 µM; HepG2: 100, 250 and 500 µM) and IMZ (Caco-2: 50, 75, 250 µM; HepG2: 25, 50 and 75 µM) diluted in FBS-free culture media. Negative control cells (non-exposed cells) were included in each assay. After a 24 h incubation with test solutions, cells were washed with phosphate buffer saline (PBS) solution and then detached from the microplates (as in Section 3.2.1) and placed in centrifuge tubes and centrifuged (5 min, 3000 rpm, bench micro-centrifuge). After eliminating the supernatant, cells were washed in PBS solution through another centrifuge cycle. Then cells were divided before being incubated with the specific markers to assess oxidative stress markers and cell apoptosis/necrosis. 

#### 3.3.2. Assessment of Oxidative Stress Induced by Pesticides

To assess pesticides-induced oxidative stress, the intracellular content in reactive oxygen species (ROS), content in glutathione (GSH), and lipid peroxidation was quantified by flow cytometry, following the methods described by Domínguez-Perles, et al. [41] and Queiroz, et al. [69]. Briefly, to assess ROS content, cells re-suspended in PBS (from Section 3.3.1) were centrifuged (5 min, 3000 rpm, bench micro-centrifuge) and, after eliminating the supernatant, 200 μL of 10 µM DCFDA solution (prepared in FBS-free DMEM) was added to each sample. After a 45 min incubation at 37 °C (in the dark, in an incubator), cells were centrifuged to remove excess of probe and re-suspended in PBS. Finally, 5 μL of propidium iodide (PI; 50 μg/mL) was added to each sample 3 min before flow cytometry acquisition.

To assess GSH content, cells re-suspended in PBS (see Section 3.3.1) were incubated with 40 μM Mercury Orange (1-(4-chloromercuryphenyl-azo-2-naphtol) for 5 min (in the dark, room temperature), as described by du Plessis, et al. [40].

Lipid peroxidation was evaluated using the DHPE-FITC probe. Cells re-suspended in PBS (see Section 3.3.1) were incubated with 20 μM DHPE-FITC, for 20 min in the dark at room temperature (as describe [41,42]). 

#### 3.3.3. Evaluation of Pesticide-Induced Mitochondrial Membrane Depolarization

Mitochondrial membrane potential was analysed using JC-1 probe following the procedures as described by the manufacturer. Caco-2 and HepG2 cells suspended in PBS were centrifuged (5 min, 3000 rpm, bench micro-centrifuge), the supernatant was removed and 200 μL of JC-1 solution (2 μM; diluted in FBS-free culture media) was added to each sample. The samples were incubated for 20 min at 37 °C (in a CO_2_ incubator) and then analysed by flow cytometry. Negative control, non-exposed, and positive control cells exposed to the protonophore FCCP (5 μM; carbonyl cyanide *p*-trifluoromethoxyphenylhydrazone) were performed in each set of experiments.

#### 3.3.4. Evaluation of Pesticide-Induced Apoptosis and Necrosis

Flow cytometry analysis of cell apoptosis was performed by double incubation with annexin-V/PI, as described by Martins-Gomes, et al. [36] and Domínguez-Perles, et al. [41].Caco-2 and HepG2 cells (see Section 3.3.1) suspended in PBS were centrifuged (5 min, 3000 rpm, bench micro-centrifuge), then supernatants were removed and 200 μL of annexin-V-FITC solution (dilution 1:200; in annexin-V binding buffer (pH 7.4): 10 mM HEPES sodium salt, 150 mM NaCl, 5 mM KCl, 5 mM MgCl_2_ and 1.8 mM CaC1_2_) was added and cells were gently suspended in this solution. The samples where then incubated for 20 min at room temperature. After this period, 5 μL of propidium iodide (PI; at 50 μg/mL) solution were added to each sample 3 min before acquisition.

### 3.4. Data and Statistical Analysis

Data are presented as mean ± SD (*n* = 4 independent assays for cell viability assay and *n* = 3 for flow cytometry analysis). The IC_50_ values, calculated from the for cell viability/toxicity assay were calculated as described by Silva, et al. [68]. Significant differences between samples and the control were performed using the t-Student test (α = 0.05). For the comparison of the IC_50_ values, an analysis of variance (ANOVA) followed by Tukey’s multiple test (α = 0.05) was performed using the tolls of GraphPad Prism version 7 (GraphPad Software Inc., San Diego, CA, USA).

## 4. Conclusions

In the present study we provide a comparison between the toxicity of three worldwide used pesticides, namely an herbicide (glyphosate), an insecticide (imidacloprid), and a fungicide (imazalil) and their effect on Caco-2 and HepG2 cells aiming to study the toxicity effect of oral exposure, either accidental or as food contaminants. Pesticides showed a rank of toxicity, GLY < IMD < IMZ, identical to both cell lines, although toxicity in HepG2 cells was higher than in Caco-2 cells. Mechanisms of toxicity involve increase in ROS, loss of mitochondrial membrane potential, and apoptotic events. Toxicity is correlated with the pesticides’ physicochemical parameters, such as the Log P_ow_ (*n*-octanol/water partition coefficient), which is directly proportional with toxicity, while a decrease in TPSA and in H-bond are likely to be directly correlated with pesticide-induced toxicity. Thus, pesticides’ physicochemical parameters that may infer about pesticides potential to interact with cell membranes or to permeate them constitute key factors to predict the toxicity of xenobiotics. In this work we show for the first time the comparison of three distinct pesticides concerning their toxicological effect on Caco-2 and HepG2 cells at oxidative stress and apoptosis level and the correlation of the toxicological obtained results with the physicochemical parameters of pesticides. Molecular physicochemical parameters are potential good predictors of toxicity and can be applied to a plethora of molecules. 

## Figures and Tables

**Table 1:** IC_50_ values of pesticide-induced cytotoxicity in Caco-2 and HepG2 cells.

	IC_50_ (μM)					
	Caco-2			HepG2		
	24 h	48 h		24 h	48 h	
Glyphosate	>1000	>1000	n.s.	>1000	>1000	n.s.
Imidacloprid	>1000	832 ± 30	*	624 ± 24	620 ±11	n.s.
Imazalil	254 ± 3	187 ± 2	*	94 ± 12	47 ± 3	*

Notes: Values are presented as mean ± SD (*n* = 4). Differences between exposure times were considered significant when *p* < 0.05, and denoted as “*”; n.s.—not significant.

**Table 2:** Concentrations of glyphosate (GLY), imidacloprid (IMD) and imazalil (IMZ) chosen to proceed with the flow cytometry assays based on Caco-2 and HepG2 cells toxicity results.

	Caco-2			HepG2		
GLY (μM)	250	500	750	250	500	750
IMD (μM)	250	500	750 (^1^)	100	250	500 (^2^)
IMZ (μM)	50	75	250 (^3^)	25	50	75 (^4^)

^1^ Value close (slightly below) to IMD IC_50_ in Caco-2 cells. ^2^ Value close (slightly below) to IMD IC_50_ in HepG2 cells. ^3^ Value of IMZ IC_50_ in Caco-2 cells. ^4^ Value close (slightly below) to IMZ IC_50_ in HepG2 cells.

**Table 3:** Physicochemical properties of glyphosate, imidacloprid and imazalil considered relevant to their toxicity against Caco-2 and HepG2 cells. Data were obtained from Pubchem and INCHEM (IPCS, World Health Organization) [47,48,49,50].

Physicochemical Parameter	Glyphosate	Imidacloprid	Imazalil
Molecular weight (MW)	169.07	255.66	297.2
H-bond (donor count)	4	1	0
H-donor (acceptor count)	6	4	2
Rotatable bound count	4	2	6
Water solubility (g/L)	12.0	0.6	1.4
Partition Coefficient *n*-octanol/water (Log P_ow_)	−1.0	0.57	4.56
Topological polar surface area (TPSA) index (Å^2^)	107	86.3	27

## Data Availability

Not applicable.
